# Author Correction: Cryo-EM structures of thermostabilized prestin provide mechanistic insights underlying outer hair cell electromotility

**DOI:** 10.1038/s41467-022-34985-0

**Published:** 2022-11-28

**Authors:** Haon Futamata, Masahiro Fukuda, Rie Umeda, Keitaro Yamashita, Atsuhiro Tomita, Satoe Takahashi, Takafumi Shikakura, Shigehiko Hayashi, Tsukasa Kusakizako, Tomohiro Nishizawa, Kazuaki Homma, Osamu Nureki

**Affiliations:** 1 Department of Biological Sciences, Graduate School of Science, The University of Tokyo, Bunkyo-ku, Tokyo, 113-0033 Japan; 2 Department of Otolaryngology—Head and Neck Surgery, Feinberg School of Medicine, Northwestern University, Chicago, IL 60611 USA; 3 Department of Chemistry, Graduate School of Science, Kyoto University, Kitashirakawa, Oiwake-cho, Sakyo-ku, Kyoto, 606-8502 Japan; 4 The Hugh Knowles Center for Clinical and Basic Science in Hearing and Its Disorders, Northwestern University, Evanston, IL 60608 USA; 5 Present Address: Department of Life Sciences, Graduate School of Arts and Sciences, The University of Tokyo; Meguro-ku, Tokyo, 153-8503 Japan; 6 Present Address: MRC Laboratory of Molecular Biology, Cambridge, UK; 7 Present Address: Graduate School of Medical Life Science, Yokohama City University, Yokohama, Japan

**Keywords:** Cryoelectron microscopy, Hair cell, Structural biology

Correction to: *Nature Communications* 10.1038/s41467-022-34017-x, published online 20 October 2022

The original version of this Article contained errors in the Results, Discussion and References sections.

The incorrect sentence in the Results section read ‘The TM domain is divided into two subdomains: the core (TM helices 1–4 and 9–11) and the gate (TM helices 5–8 and 12–14) domains (Fig. 1c, d).’ The correct version states ‘(TM helices 1–4 and 8–11) and the gate (TM helices 5–7 and 12–14)’ in place of ‘(TM helices 1–4 and 9–11) and the gate (TM helices 5–8 and 12–14)’.

The incorrect sentence in the Discussion section read ‘The cryo-EM structure of PresTS suggested highly stable dimer formation, mediated by hydrophobic residues at the C-terminal ends of TM14 (Fig. 6a), and multiple lipids filling the dimeric junction region (Fig. 6c, d).’ The correct version states ‘(Fig. 6c)’ in place of ‘(Fig. 6c, d)’.

The original version of this Article contained an error in Ref. 60, which directed to a preprint ‘Yamashita, K., Palmer, C. M., Burnley, T. & Murshudov, G. N. Cryo-EM single particle structure refinement and map calculation using Servalcat. *bioRxiv* 10.1101/2021.05.04.442493 (2021)’. The correct form of Ref. 60 is: ‘Yamashita, K., Palmer, C. M., Burnley, T. & Murshudov, G. N. Cryo-EM single particle structure refinement and map calculation using Servalcat. *Acta Crystallogr. Sect. D Struct. Biol*. **77**, 1282–1291 (2021)’.

These have been corrected in both the PDF and HTML versions of the Article.

