# Supplementary material for: Cryo-EM structures of thermostabilized prestin provide mechanistic insights underlying outer hair cell electromotility
Source: Nat Commun. 2022 Oct 20;13:6208. doi: 10.1038/s41467-022-34017-x (PMC9584906; doi:10.1038/s41467-022-34017-x)
Supplement: Supplementary file 1 — Supplementary Information [file 41467_2022_34017_MOESM1_ESM.pdf]

Supplementary Information for

Cryo-EM structures of thermostabilized prestin provide mechanistic insights underlying outer hair cell electromotility

H. Futamata, M. Fukuda, R. Umeda, K. Yamashita, A. Tomita, S. Takahashi, T. Shikakura, S. Hayashi, T. Kusakizako, T. Nishizawa, K. Homma and O. Nureki.

Correspondence to: k-homma@northwestern.edu (K.H.), t-2438@yokohama-cu.ac.jp (T.N.) and nureki@bs.s.u-tokyo.ac.jp (O.N.)

This PDF file includes:

Supplementary Figs. 1 to 16
Supplementary Table 1
Captions for Supplementary Data 1 and 2, Movies 1, 2 and 3
Supplementary References

Other Supplementary Materials for this manuscript include the following:

Supplementary Movies 1, 2 and 3
Supplementary Data 1 and 2

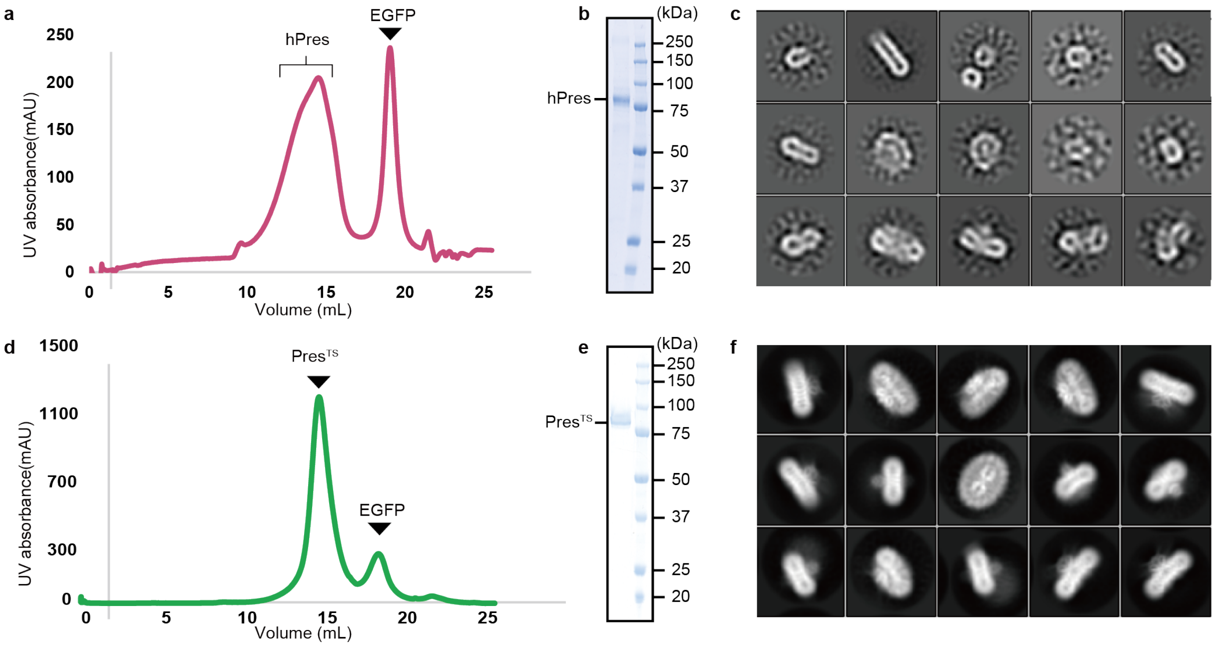

Supplementary Fig. 1. Purification of wild-type hPres and Pres^{TS}.

(a) SEC profile of hPres. The elution fractions of hPres and EGFP are indicated. **(b)** SDS-PAGE analysis of the peak fraction corresponding to hPres. We repeated twice, and the panel shows the representative result. **(c)** Representative averaged 2D classes of wild-type hPres, indicating the high heterogeneity of the purified hPres protein. **(d)** SEC profile of Pres^{TS}. The elution fractions of Pres^{TS} and EGFP are indicated. **(e)** SDS-PAGE analysis of the peak fraction corresponding to Pres^{TS}. We repeated twice, and the panel shows the representative result. **(f)** Representative averaged 2D classes of Pres^{TS}.

a

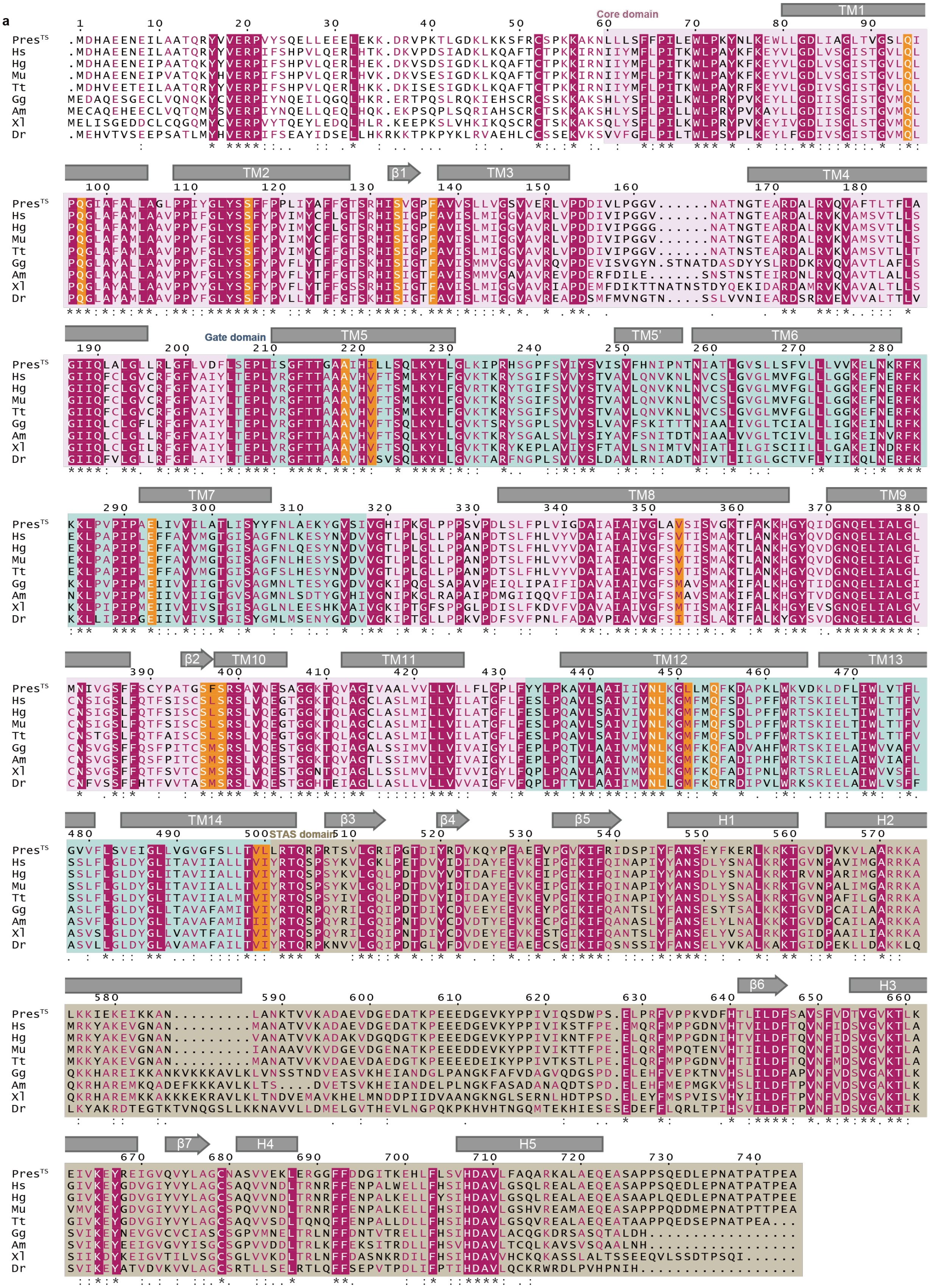

Core domain
Gate domain
STAS domain
TM1
TM2
TM3
TM4
TM5
TM5'
TM6
TM7
TM8
TM9
TM10
TM11
TM12
TM13
TM14
β1
β2
β3
β4
β5
β6
β7
H1
H2
H3
H4
H5
Pres[TS]
Hs
Hg
Mu
Tt
Gg
Am
Xl
Dr

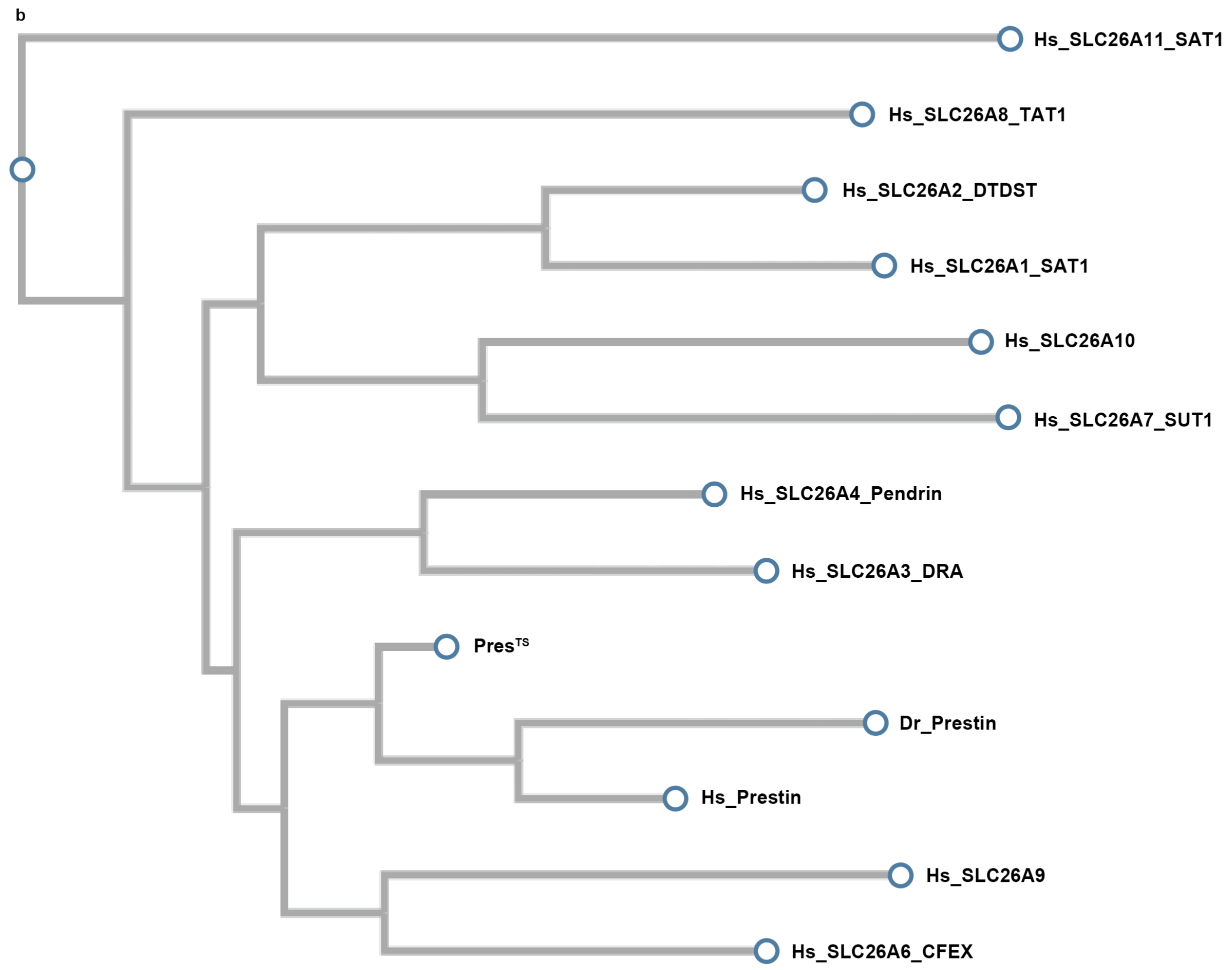

Supplementary Fig. 2. Sequence alignment of Pres^{TS} and prestin orthologs.

(**a**) Sequence comparison of Pres^{TS} and representative prestin orthologs. The sequences are Pres^{TS}, human (Hs), UniProt: P58743; naked mole-rat (Hg), UniProt: Q99NH7; mouse (Mu), UniProt: D7PC76; Atlantic bottle-nosed dolphin (Tt), UniProt: G5BI49; chicken (Gg), UniProt: A0A151NPE2; Alligator mississippiensis (Am), UniProt: A0A1L8GTW4; African clawed frog (Xl), UniProt: A0FKN5; zebrafish (Dr), UniProt: Q7T2N6. The locations of the transmembrane helices (TM), other α helices (H), and β strands (β) are indicated. See also Supplementary Data 1 for the frequency of each residue. The residues contributing to anion binding, the core-gate domain interface, and the dimerization interface within the transmembrane region are highlighted in orange. The alignment was performed by ClustalW[1,2] and is displayed with consensus symbols: "* (asterisk)" identical in all sequences, ": (colon)" weakly conserved, ". (period)" highly conserved as similar residues (i.e., E to D). The figure was created with the ESPript 3.0 web tool (https://espript.ibcp.fr/ESPript/cgi-bin/ESPript.cgi)[3]. (**b**) A phylogenetic tree constructed for prestin and other SLC26 family proteins: Pres^{TS}, human (Hs) SLC26A1, SLC26A2, SLC26A3, SLC26A4, SLC26A5, SLC26A6, SLC26A7, SLC26A8, SLC26A9, SLC26A10, SLC26A11, and zebrafish (Dr) SLC26A5, using FastTree[4,5] with the default parameters.

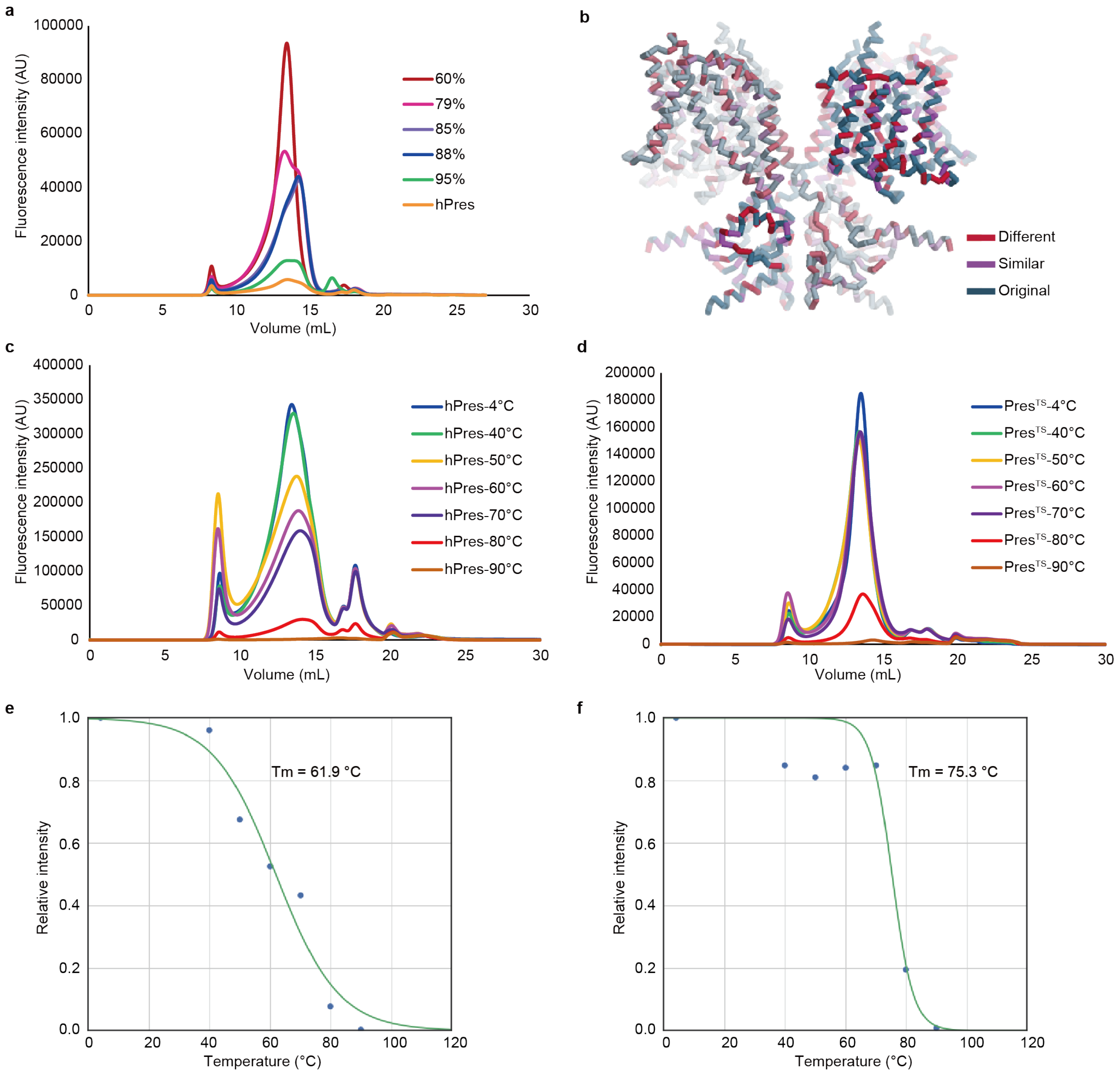

Supplementary Fig. 3. FSEC traces and thermostabilities of prestin and its mutant constructs.

(**a**) FSEC chromatogram of hPres and its thermostabilized prestin mutants detected by GFP fluorescence. The labels indicate the threshold of conservation. For example, 60% indicates that the amino acid sequences that are less than 60 % conserved are mutated to the most conserved one among species (see Methods). The prestin construct with “60% threshold” was the most stable as the dimer when solubilized with DDM. We further reverted a few residues that are presumed to be important for anion binding, and the resultant construct was named Pres^{TS} and used for structural analysis. (**b**) The TS mutations mapped on the cryo-EM structure of Pres^{TS}. Residues mutated to those with different or similar properties are colored red and purple, respectively. The non-mutated amino acids are colored dark green. FSEC-TS chromatograms of hPres (**c**) and Pres^{TS} (**d**). The thermo-stability of prestin was determined by applying thermal shocks from 4 to 90°C. Pres^{TS} showed greater thermo-stability as compared to wild-type hPres. The T_m values were estimated to be 61.9°C and 75.3°C for hPres (**e**) and Pres^{TS} (**f**), respectively.

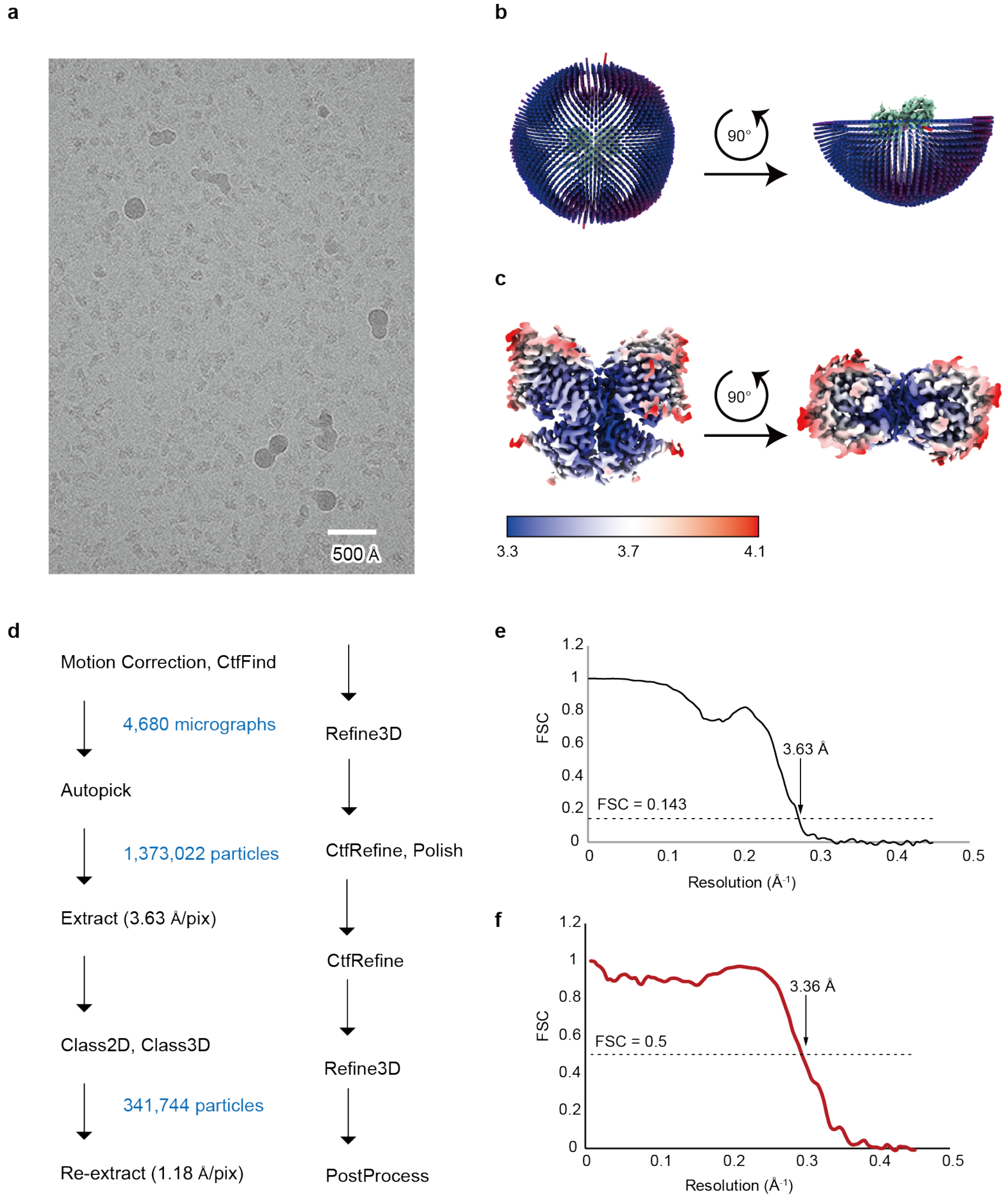

Supplementary Fig. 4. Data processing of the Cl^--bound structure of $Pres^{TS}$.

(a) Representative cryo-EM micrograph of the structure of $Pres^{TS}$ in complex with Cl^-, recorded on a 300 kV Titan Krios electron microscope with a K3 camera. We made 2 vitrified grids, and the micrographs were collected from a single grid. **(b)** Angular distribution plot of the particles used for the final 3D reconstruction. The distribution was calculated with RELION-3.1. The color and height (from blue to red) of the cylinder bars are proportional to the number of particles in those views. **(c)** Local resolution map of the prestin model calculated with RELION-3.1. **(d)** Data processing workflow of the single particle image processing. **(e)** Fourier Shell Correlation (FSC) between the two independently refined half-maps. **(f)** FSC curves for map-to-model fitting.

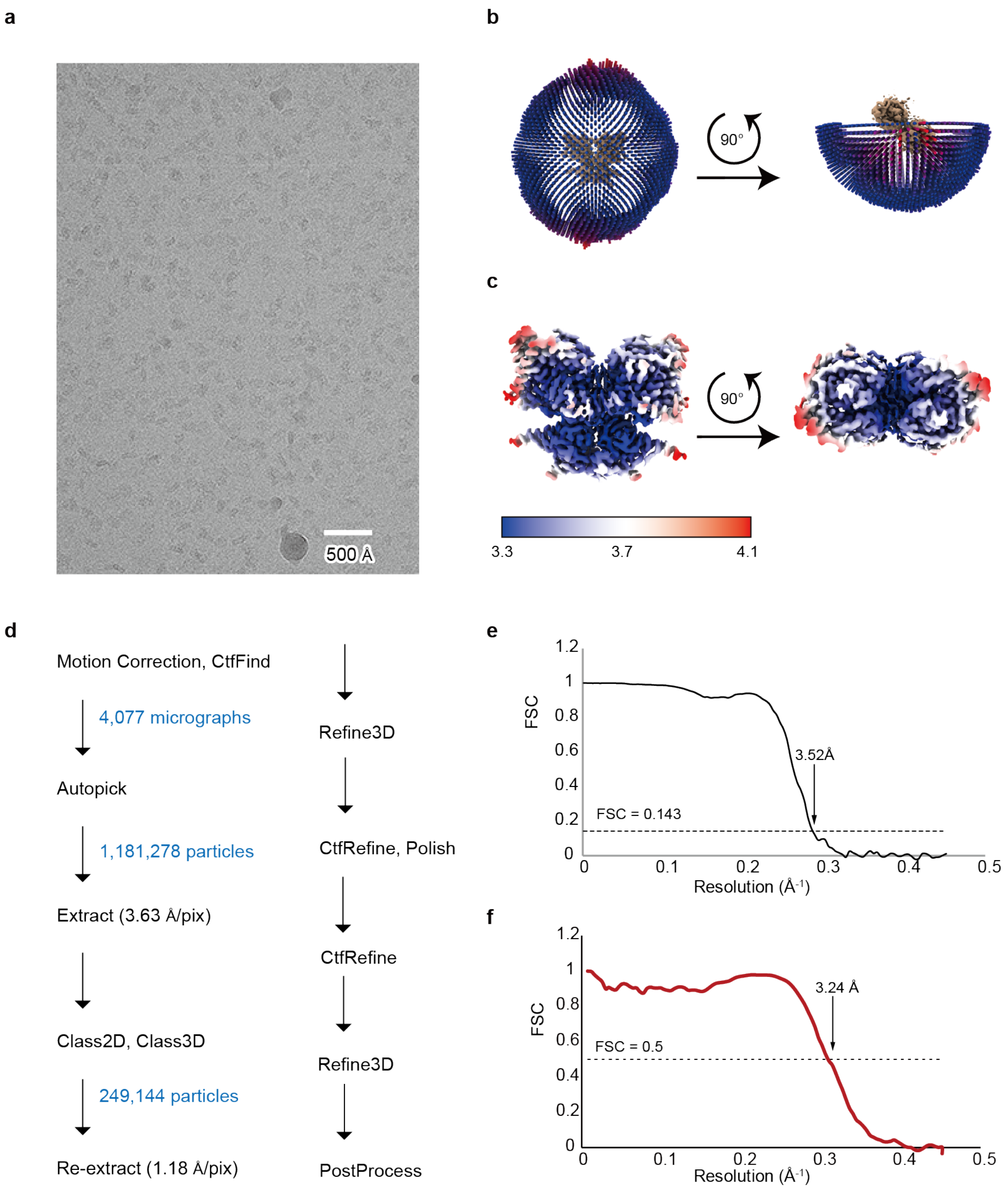

Supplementary Fig. 5. Data processing of the SO_4^{2-}-bound structure of Pres^{TS}.

(a) Representative cryo-EM micrograph of the structure of Pres^{TS} in complex with SO_4^{2-}, recorded on a 300 kV Titan Krios electron microscope with a K3 camera. We made 2 vitrified grids, and the micrographs were collected from a single grid. **(b)** Angular distribution plot of the particles used for the final 3D reconstruction. The distribution was calculated with RELION-3.1. The color and height (from blue to red) of the cylinder bars are proportional to the number of particles in those views. **(c)** Local resolution map of the prestin model calculated with RELION-3.1. **(d)** Data processing workflow of the single particle image processing. **(e)** FSC between the two independently refined half-maps. **(f)** FSC curves for map-to-model fitting.

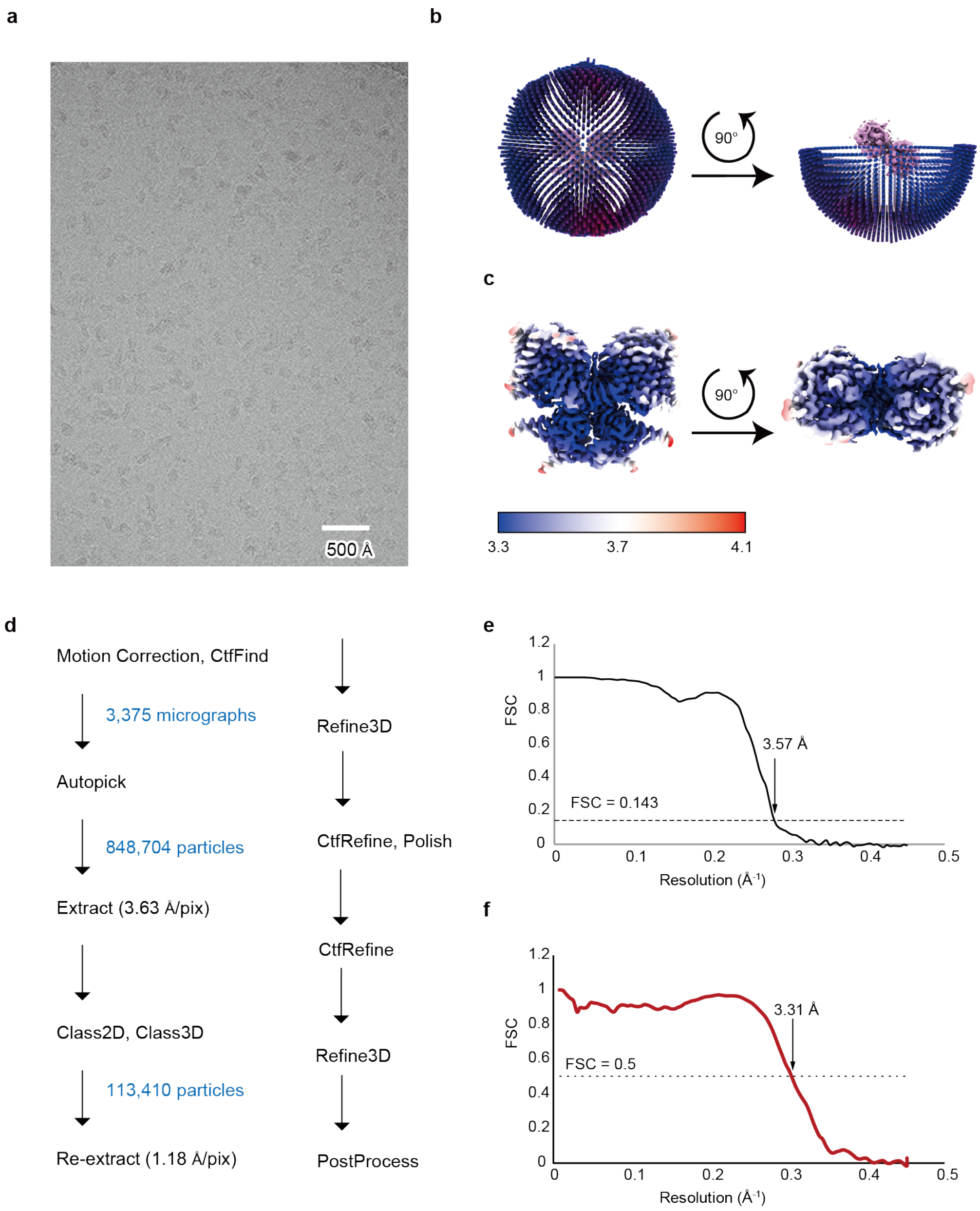

Supplementary Fig. 6. Data processing of the salicylate-bound structure of Pres^{TS}.

(a) Representative cryo-EM micrograph of the structure of Pres^{TS} in complex with salicylate, recorded on a 300 kV Titan Krios electron microscope with a K3 camera. We made 2 vitrified grids, and the micrographs were collected from a single grid. **(b)** Angular distribution plot of the particles used for the final 3D reconstruction. The distribution was calculated with RELION-3.1. The color and height (from blue to red) of the cylinder bars are proportional to the number of particles in those views. **(c)** Local resolution map of the prestin model calculated with RELION-3.1. **(d)** Data processing workflow of the single particle image processing. **(e)** FSC between the two independently refined half-maps. **(f)** FSC curves for map-to-model fitting.

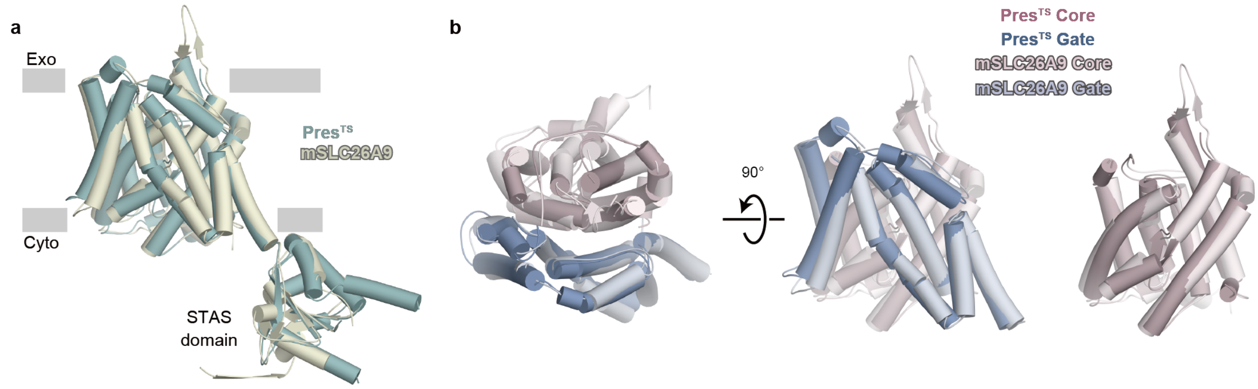

Supplementary Fig. 7. Structural comparison of Pres^{TS} and mSLC26A9.

(a) Structural superimposition of protomers of Pres^{TS} (green) and murine SLC26A9 (mSLC26A9) (PDBID: 6RTC, light beige). Both molecules are shown as cylinder models. Residues 58-722 of hPres^{TS} and 5-740 of murine SLC26A9 are superimposed (R.m.s. deviation is 1.66 Å). Gray boxes indicate the location of the lipid bilayer, with extracellular (Exo) and cytosolic (Cyto) sides as shown. **(b)** Structural superimposition of the transmembrane domains of Pres^{TS} (the same color scheme as in Fig. 1c) and the core (light pink) and gate (light blue) domains of murine SLC26A9 viewed from the extracellular side (left), the gate domain viewed from the lateral side (middle) and the core domain viewed from the lateral side (right).

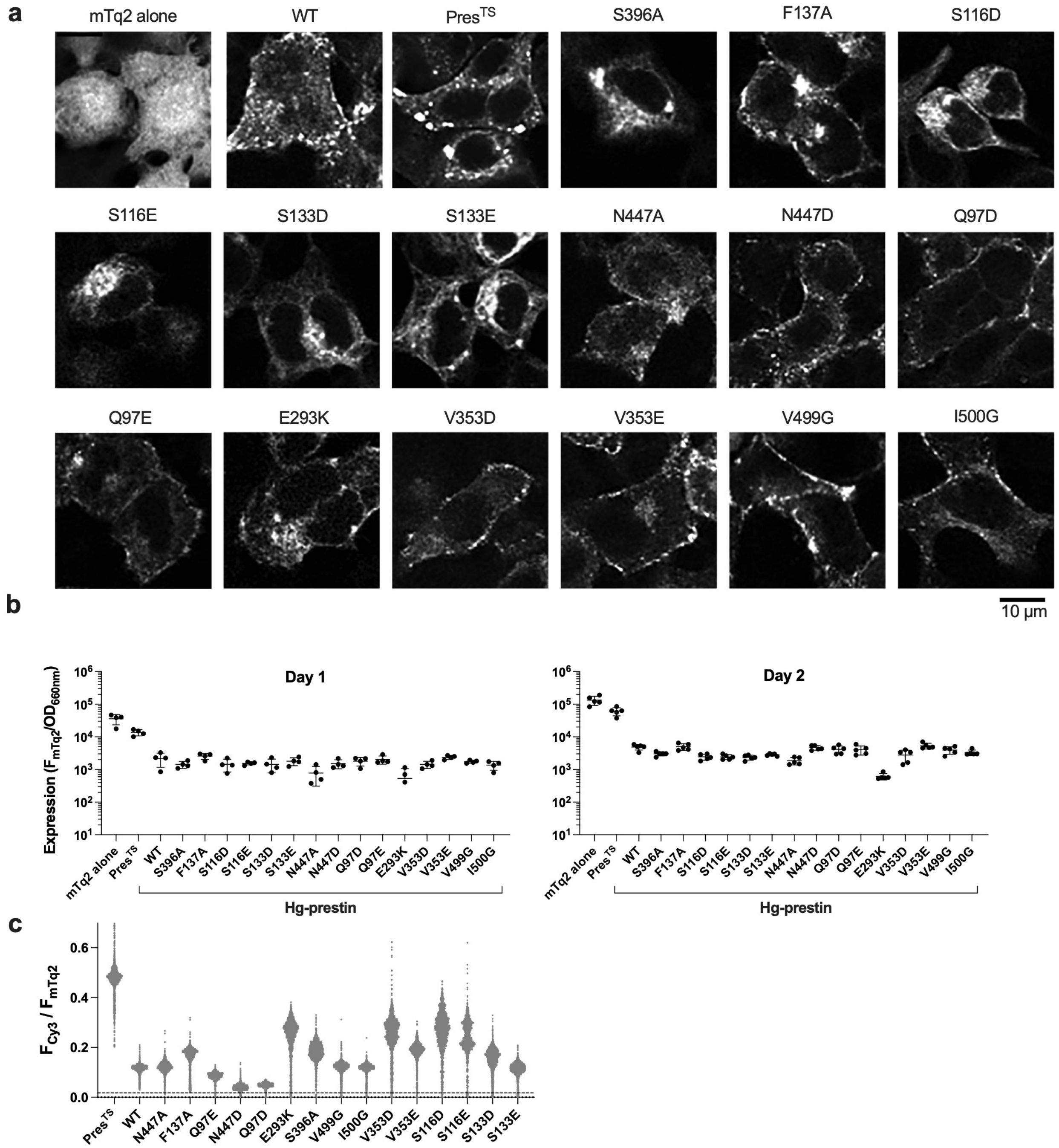

Supplementary Fig. 8. Expressions of the prestin constructs used in this study.

(a) Confocal images of stable cell lines expressing mTurquoise2 (mTq2)-tagged $Pres^{TS}$ and HgPres variants. Cells were fixed and imaged after two days of 1μg/ml doxycycline treatment (0.1 μg/ml for control cells expressing mTq2 alone). We conducted four experiments for each mutant, and the figure shows the representative images. **(b)** Total expression levels of mTq2-tagged $Pres^{TS}$ and HgPres variants after one day (left) and two days (right) in 1μg/ml doxycycline (0.1 μg/ml for control cells expressing mTq2 alone). Collected cells were suspended in PBS at each time point and transferred to a 96-well plate for plate reader measurement of

mTq2 fluorescence. Expression levels were normalized using the optical density measured at 660 nm. Individual data points indicate independent experiments. Horizontal bars indicate mean ± SD. **(c)** Membrane targeting efficiencies of the prestin constructs. mTq2-tagged prestin constructs expressed in HEK293T cells were labeled with a membrane impermeable fluorescent probe, sulfo-Cy3 NHS ester. The cells were lysed, and the detergent extracted mTq2-tagged prestin proteins were captured by anti-mTq2 affinity beads. Fluorescence intensities in the cyan (F_{mTq2}) and red (F_{Cy3}) channels were determined from images of the beads, and their ratios (F_{Cy3}/F_{mTq2}) were determined. The horizontal dashed line indicates the mean F_{Cy3}/F_{mTq2} value of negative control (mTq2 alone). Two independent experiments were performed to collect these data.

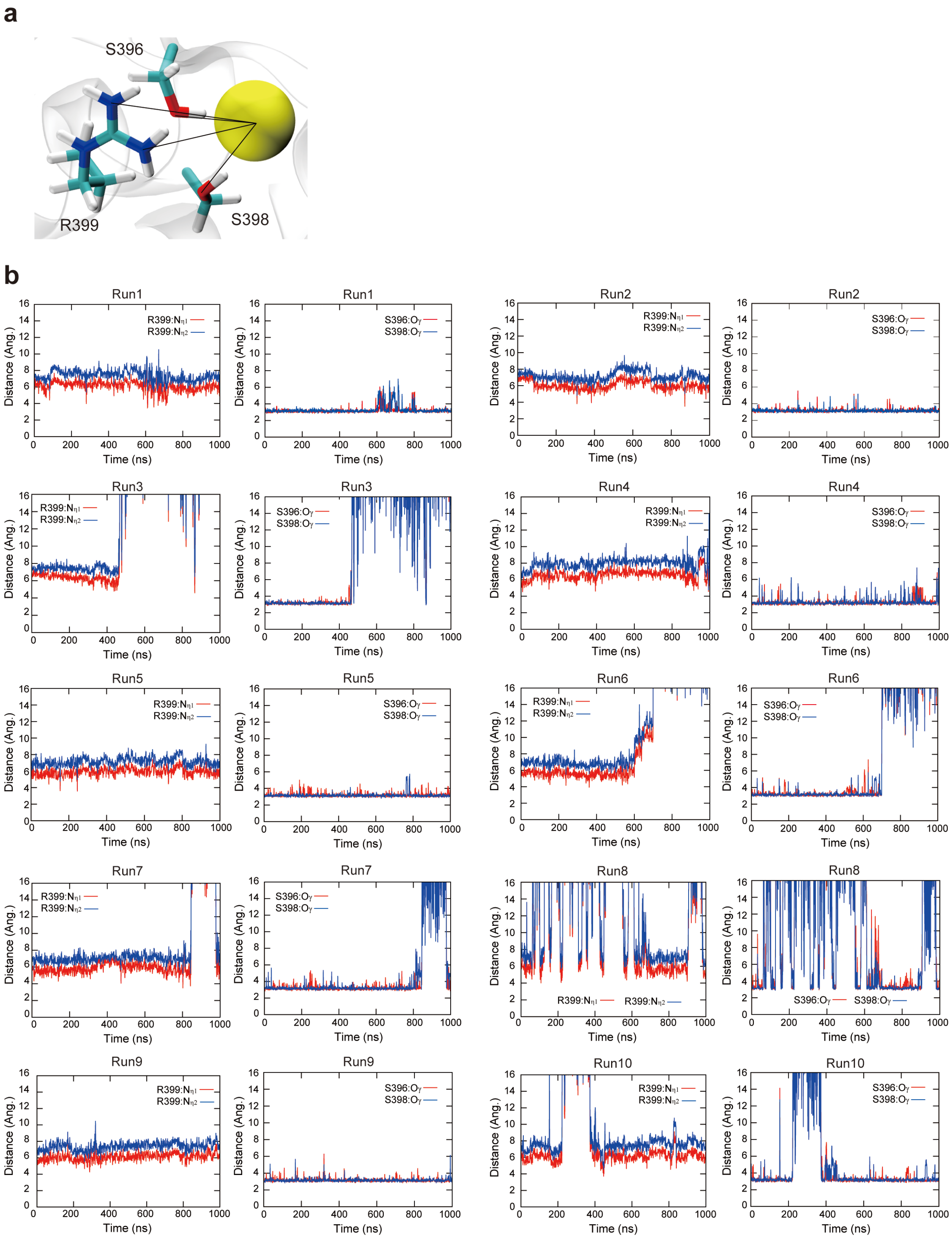
a
S396
R399
S398
b
Run1
Run2
Run3
Run4
Run5
Run6
Run7
Run8
Run9
Run10
R399:Nη1
R399:Nη2
S396:Oγ
S398:Oγ
Distance (Ang.)
Time (ns)

Supplementary Fig. 9. Stability of Cl^- ion binding at the binding site in MD simulations.

(a) The chloride binding site. A Cl^- ion (yellow sphere) and interacting groups in the vicinity of the Cl^- ions are depicted. Measured distances are indicated by lines. (**b**) Temporal changes of the distances between the closest Cl^- ion and the interacting groups in 10 independent 1 μs MD trajectories. The distance between the Cl^- ion and the closest N_η atom of R399 is distributed in 5-7 Å when the Cl^- ion is bound, showing that the Cl^- ion and R399 do not form a direct contact as described in the main text. The distance is somewhat larger than that observed in the cryo-EM $Pres^{TS}$ structure, 4.49 Å, presumably because of thermal fluctuation in the MD simulations at 300 K and the use of the native protein structure modeled from the $Pres^{TS}$ one.

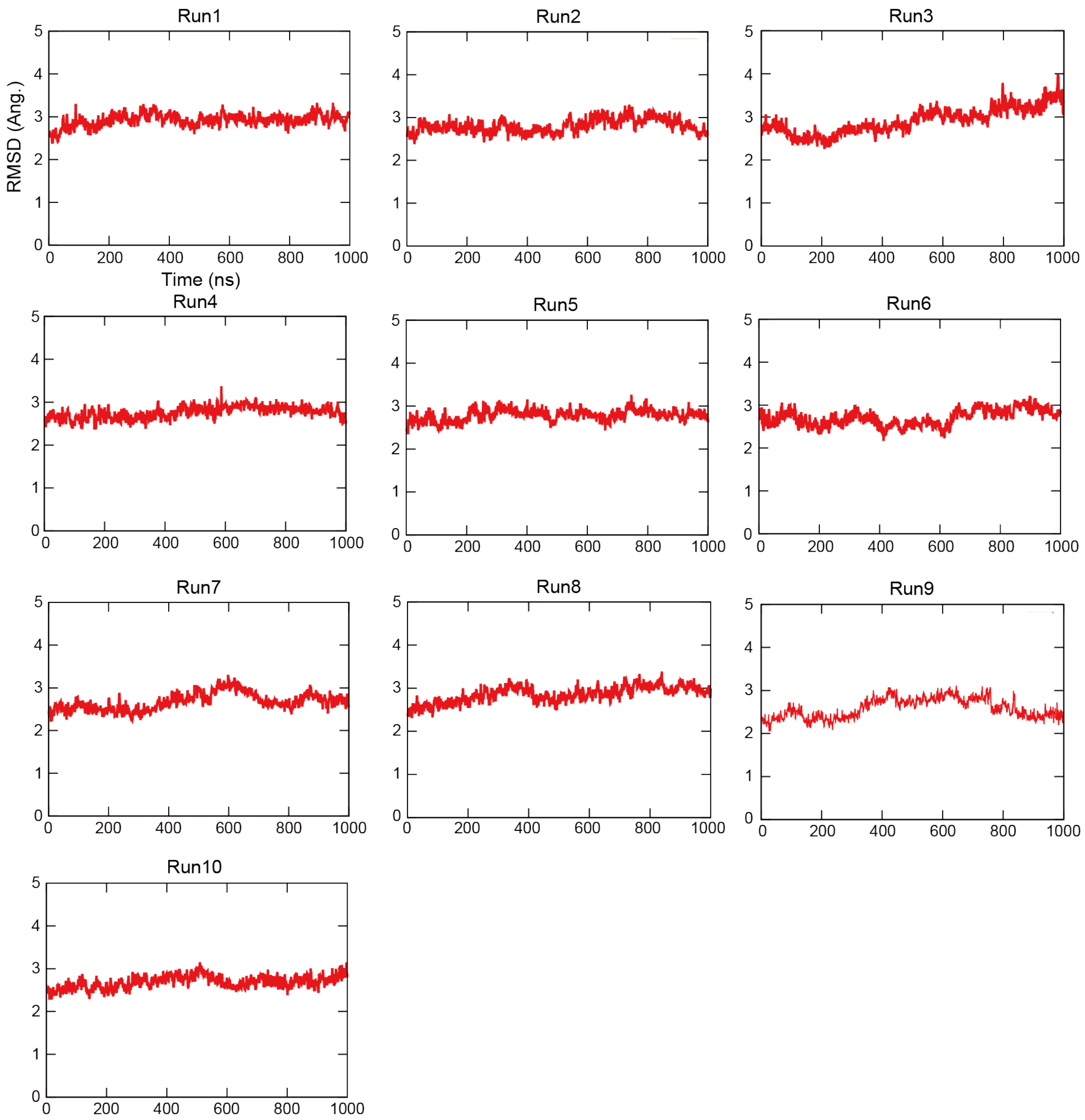

Supplementary Fig. 10. Temporal changes of RMSDs of the transmembrane domain (Arg58 to Pro506) of 10 independent 1 μs MD trajectories with respect to that of the cryo-EM structure.

The protein conformations in the Cl^- ion bound state remained stable during each 1 μs simulation, while slight increases of the RMSD upon the release of the Cl^- ion (e.g. Run3) were observed.

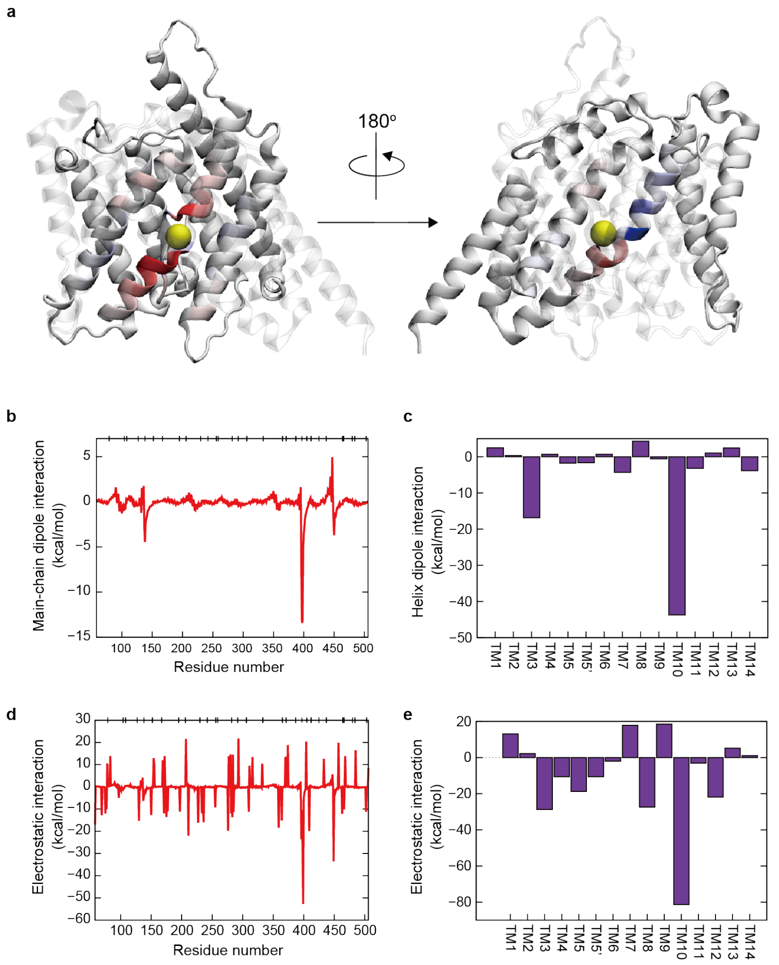

Supplementary Fig. 11. Electrostatic interactions between the bound Cl^- ion and the protein.

(**a**) Heatmaps of the interaction energies with the dipole moments of the main-chain parts of the individual amino acids shown in (**b**). Contributions of the core domain (left) and the gate domain (right) are depicted. (**b**) Interaction energies of a bound Cl^- ion with dipole moments of the main-chain parts of the individual amino acids. (**c**) Interaction energies of a bound Cl^- ion with dipole moments of the mainchains of transmembrane helices. The amino acids included in the transmembrane helices are defined in Supplementary Fig. 2, and their contributions are accumulated for the interaction energies of transmembrane helices. (**d**) Total electrostatic interaction energies of a bound Cl^- ion with the individual amino acids. (**e**) Total electrostatic interaction energies of a bound Cl^- ion with the amino acids in the individual transmembrane helices. Note that the total electrostatic interaction energies include contributions of the interaction energies with the dipole moments of the main-chain parts shown in (**b)** and (**c)**. For TM10, therefore, the total electrostatic interaction of −81 kcal/mol is composed of the helix dipole interaction energy of −44 kcal/mol and the other electrostatic interactions, in which a major contribution is the electrostatic interaction with the positively charged sidechain of R399. Together with the large contribution of the helix dipole interaction of TM3, −17 kcal/mol, the electrostatic interaction with the helix dipole moments of TM3 and TM10 is larger than that with the sidechain of R399.

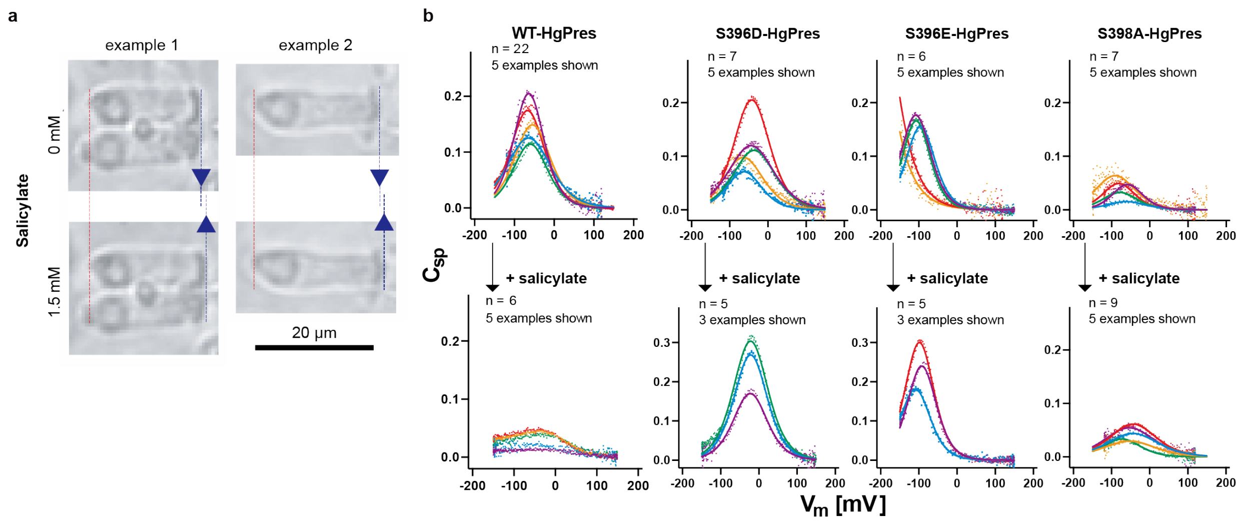

Supplementary Fig. 12. The effects of salicylate on wild-type OHCs and HEK cells expressing various HgPres constructs.

(**a**) Salicylate-induced elongation of OHCs. Video frame images of OHCs before (upper panels) and after (bottom panels) the application of 1.5 mM salicylate in the bath solution (see also Supplementary Movie 2). **(b)** The effect of salicylate on the NLC of WT-, S396D-, S396E-, and S398A-HgPres. NLC was measured in the absence (upper panels) and presence (lower panels) of 10 mM salicylate in the bath solution. Three to five examples are shown in different colors for each construct and condition.

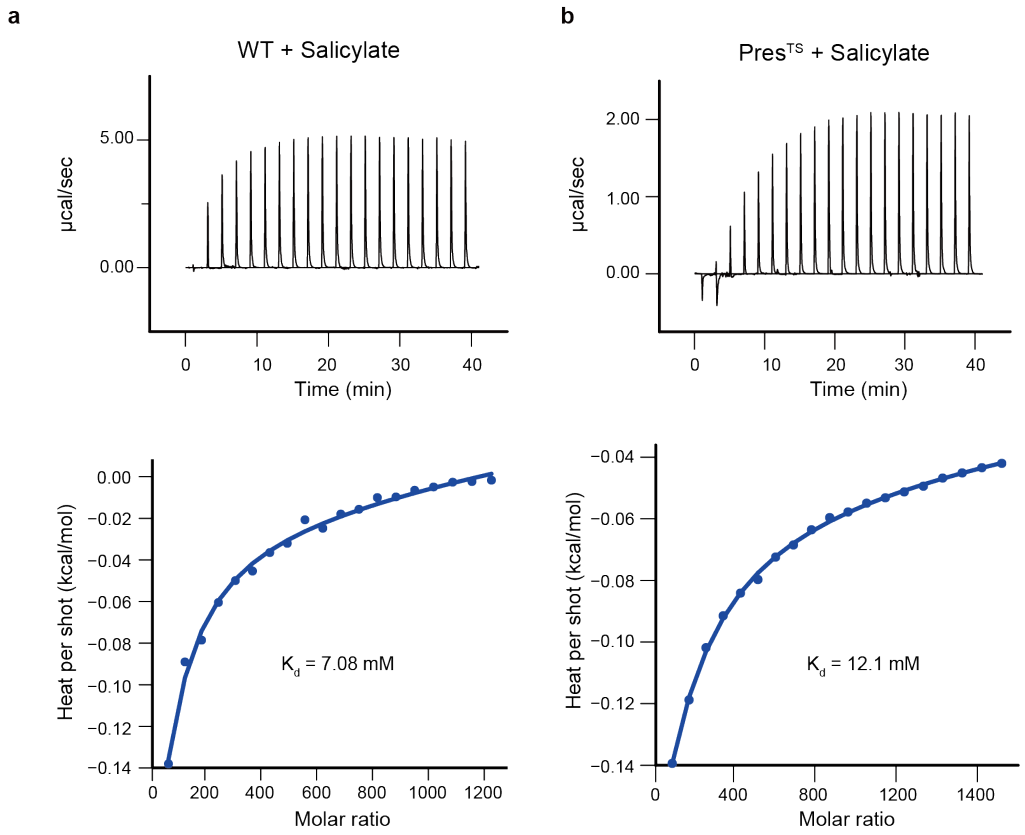

Supplementary Fig. 13. Salicylate binding to hPres and Pres^{TS}.

Salicylate binding to hPres (**a**) and Pres^{TS} (**b**) was examined by isothermal titration calorimetry (ITC). The raw ITC data (upper panel) and the plots of injected heat for 20 automatic injections of 200 mM salicylate solution into the sample cell containing hPres (WT) or Pres^{TS} (bottom panel) are shown. Measurements were repeated twice to confirm reproducibility.

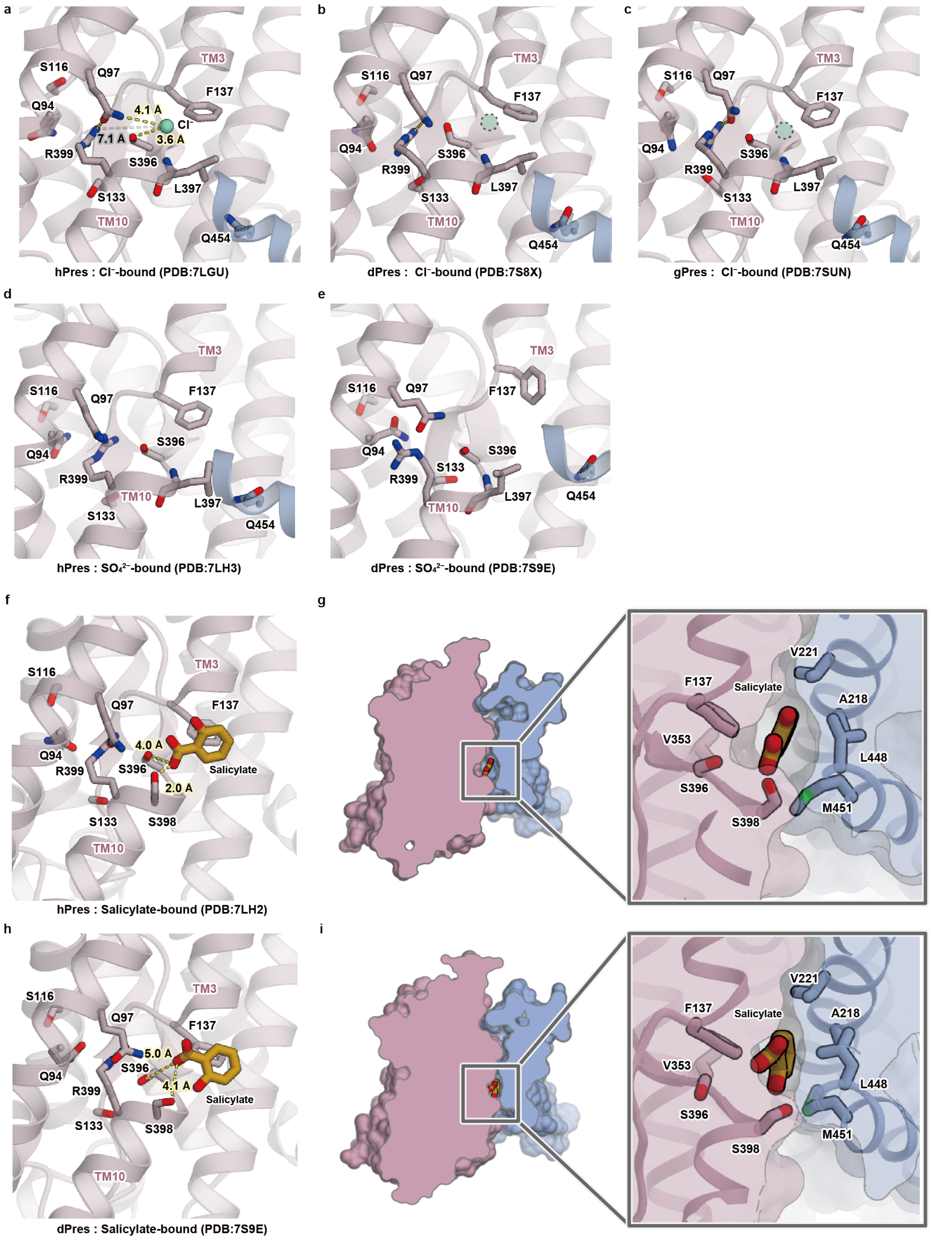

Supplementary Fig. 14. Anion binding site of hPres, dPres, and gPres.

Close-up views of the central Cl^- binding pockets of hPres (PDB: 7LGU) **(a),** dPres (PDB: 7S8X) **(b)**, and gPres (PDB: 7SUN) **(c)**. Distances for important atoms (within 5Å) are shown by yellow dotted lines. Distances over 5Å are shown by gray dotted lines. In (b) and (c), a putative Cl^- binding site is shown as a green circle within a dotted line. Close-up views of the central SO_4^{2-} binding pockets of hPres (PDB: 7LH3) **(d)** and dPres (PDB: 7S9B) **(e)**. **(f)** Close-up view of the central anion binding pocket of hPres (PDB: 7LH2) with a bound salicylate. **(g)** Salicylate sandwiched between the core and gate domains of hPres (left panel). Cose-up view of salicylate binding pocket between the core and gate domains of hPres(right panel). **(h)** Close-up view of the central anion binding pocket of dPres (PDB: 7S9E) with a bound salicylate. **(i)** Salicylate sandwiched between the core and gate domains of dPres (left panel). Close-up view of the salicylate binding pocket between the core and gate domains of dPres (right panel).

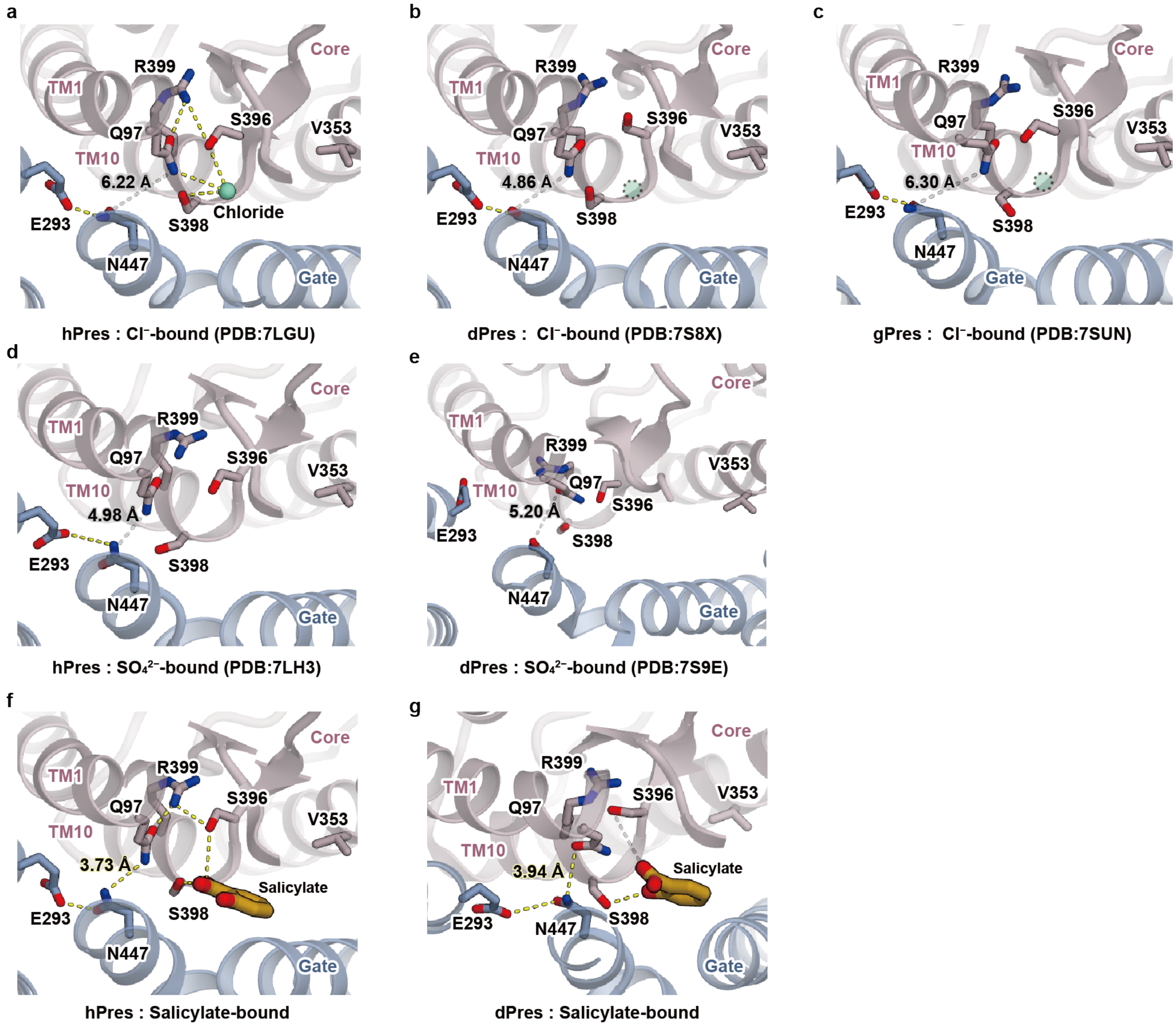

Supplementary Fig. 15. Interactions between the core and gate domains.
(a-c) The core-gate domain interfaces in the Cl^--bound hPres **(a)**, dPres **(b)**, and gPres **(c)** viewed from the extracellular side. **(d, e)** The core-gate domain interface in the SO_4^{2-}-bound hPres **(d)** and dPres **(e)**. **(f, g)** The core-gate domain interface in the salicylate-bound hPres **(f)** and dPres **(g)**. Distances for important atoms (within 4.5Å) are shown by yellow dotted lines. Distances over 4.5Å are shown by gray dotted lines. For the distance between Q97 and N447, yellow lines are shorter than 4 Å and gray lines are longer than 4 Å.

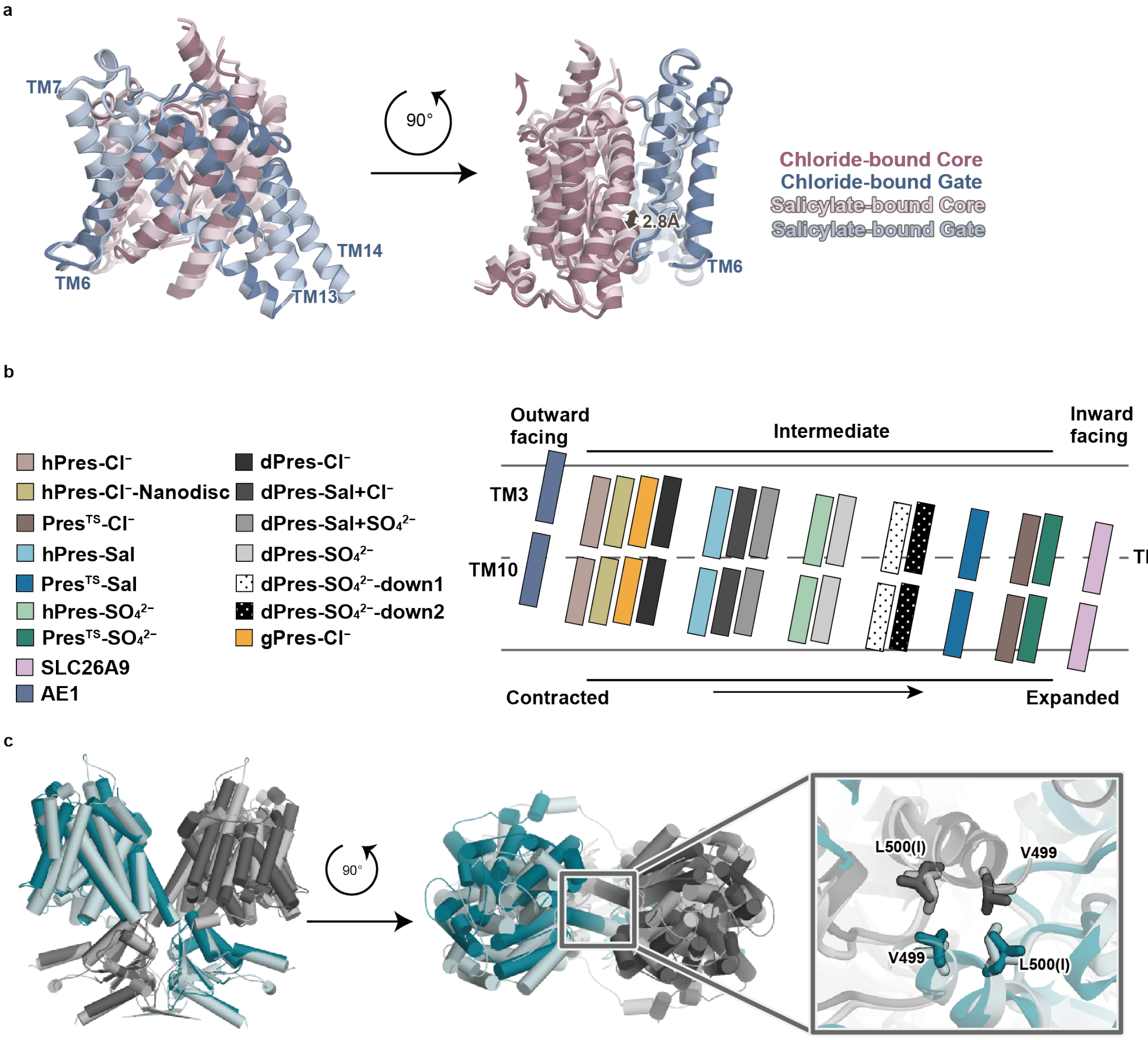

Supplementary Fig. 16. Comparisons of prestin structures.

(a) Rigid-body domain movement of the core domain with respect to the gate domain, as deduced from the Cl^{-}- vs. salicylate-bound Pres^{TS} structures (shown in dark and light colors, respectively). The distances of TM3/10 displacement in Cl^{-}- and salicylate-bound Pres^{TS} were measured using S398 of TM10 as the reference point. See also Supplementary Movie 1. **(b)** Schematic diagram showing the relative positions of TM3 and TM10 within the membrane, found in the prestin structures reported herein and by others: hPres-Cl^{-} (PDB: 7LGU), hPres-Cl^{-} in nanodisc (PDB: 7LGW), Pres^{TS}-Cl, hPres-Sal (PDB: 7LH2), Pres^{TS}-Sal, hPres-SO_4^{2-} (PDB: 7LH3), Pres^{TS}-SO_4^{2-}, SLC26A9 (PDB: 6RTC), AE1 (PDB: 4YZF), dPres-Cl^{-} (PDB: 7S8X), dPres-Salicylate-Cl^{-} (PDB: 7S9A), dPres-Salicylate-SO_4^{2-} (PDB: 7S9E), dPres-SO_4^{2-} (PDB: 7S9B, 7S9C and 7S9D), and gPres-Cl^{-} (PDB: 7SUN). The models are superimposed at the gate domain, and the TM3/10 positions are illustrated based on the centrally located S398. **(c)** Lateral view (left panel), extracellular view (center panel) and close-up extracellular view (right panel) of structural superimpositions of the dimers of Pres^{TS} (dark green/gray) and hPres (PDB:7LGU, light green/gray). Both molecules are shown as cylinder models. The models are superimposed by their dimer STAS domains (R.m.s. deviation 1.41 Å). The dimerization interface is conserved in wild-type hPres. The N-terminal structure was not observed in Pres^{TS}.

**Data collection and processing**			
EMDB-ID	31757	31758	31759
PDB ID	7V73	7V74	7V75
Bound anion	Chloride	Sulfate	Salicylate
Microscope	Titan Krios G4		
Detector	Gatan K3 Camera with Quantum LS energy filter		
Magnification	105,000		
Voltage (kV)	300		
Electron exposure (e^{-}/Å^2)	54	54	54
Defocus range (μm)	-0.8 to -1.6		
Pixel size (Å/px)	0.83		
Symmetry imposed	C2		
Number of movies	4,680	4,077	3,375
Initial particle images	1,373,022	1,181,278	848,704
Final particle images	341,744	249,144	113,410
Map resolution (Å)	3.63	3.52	3.57
FSC threshold	0.143		
Map sharpening B factor (Å^2)	-188.514	-181.17	-171.721
**Model building and refinement**			
Model composition			
Protein atoms	4620	4620	4687
Metals	0	0	0
Other atoms	252	249	299
R.M.S. deviations from ideal			
Bond lengths (Å)	0.0107	0.0124	0.012
Bond angles (˚)	1.697	2.020	1.7164
**Validation**			
Clashscore	5.78	6.79	6.41
Rotamer outliers (%)	1.99	3.58	5.68
Ramachandran plot			
Favored (%)	95.26	92.89	94.66
Allowed (%)	4.74	6.94	5.18
Outlier (%)	0	0.17	0.17

Supplementary Table 1. Data collection, processing, model refinement and validation. Clashscores, rotamer outliers, and Ramachandran plots were calculated using MolProbity [6].

Supplementary Data 1. Amino acid frequencies in consensus sequence of prestin.
The table shows the amino acid sequence of WT hPres, which consists of the most conserved amino acids in all sequences used for consensus calculation, and their frequencies. The F_{WT} column shows the extents of the conserved amino acids of the wild-type sequence (shown in WT column) in all of the sequences used for the consensus calculation. The F_{Cons} column shows the frequency of the most conserved amino acids in the calculated sequences. The F_{Con}/F_{WT} column shows the ratio of the frequencies of WT and the most conserved sequence, and this value is used as the basis for determining the introduction of the thermo-stabilizing mutations: the higher the F_{Con}/F_{WT}, the greater preference to change the amino acids from WT to the most conserved sequence.

Supplementary Data 2. DNA codon frequencies in Pres^{TS}.
The codon-optimized DNA sequence of Pres^{TS}.

Supplementary Movie 1. Rigid-body domain movement deduced from the Cl^-- vs. salicylate-bound Pres^{TS} structures.
The salicylate-bound and Cl^--bound structures are overlayed with the gate domains used as superimposition references. The anion binding site is indicated by a circle. See also Fig. 3c.

Supplementary Movie 2. Salicylate-induced elongation of mouse OHCs.
Salicylate (1.5 mM) was added to the bath solution at time zero. The total filming time is 116 seconds. OHC elongation was quantified to be 3.2±0.7% (mean±SD, n=11).

Supplementary Movie 3. Shortening of OHCs after salicylate elimination.
Salicylate (1.5 mM) was perfused away starting at time zero. The total filming time is 200 seconds. OHC shortening was quantified to be -2.9±1.5% (mean±SD, n=12).

Supplementary References

1. Thompson, J. D., Higgins, D. G. & Gibson, T. J. CLUSTAL W: improving the sensitivity of progressive multiple sequence alignment through sequence weighting, position-specific gap penalties and weight matrix choice. *Nucleic Acids Res.* **22**, 4673 (1994).
2. Chenna, R. *et al.* Multiple sequence alignment with the Clustal series of programs. *Nucleic Acids Res.* **31**, 3497 (2003).
3. Gouet, P., Robert, X. & Courcelle, E. ESPript/ENDscript: extracting and rendering sequence and 3D information from atomic structures of proteins. *Nucleic Acids Res.* **31**, 3320 (2003).
4. Price, M. N., Dehal, P. S. & Arkin, A. P. FastTree: Computing Large Minimum Evolution Trees with Profiles instead of a Distance Matrix. *Mol. Biol. Evol.* **26**, 1641–1650 (2009).
5. Price, M. N., Dehal, P. S. & Arkin, A. P. FastTree 2 – Approximately Maximum-Likelihood Trees for Large Alignments. *PLoS One* **5**, e9490 (2010).
6. Davis, I. W. *et al.* MolProbity: all-atom contacts and structure validation for proteins and nucleic acids. *Nucleic Acids Res.* **35**, W375–W383 (2007).
