# Supplementary material for: Cryo-EM structures of thermostabilized prestin provide mechanistic insights underlying outer hair cell electromotility
Source: Nat Commun. 2022 Oct 20;13:6208. doi: 10.1038/s41467-022-34017-x (PMC9584906; doi:10.1038/s41467-022-34017-x)
Supplement: Supplementary file 3 — Description of Additional Supplementary Files [file 41467_2022_34017_MOESM3_ESM.pdf]

File name: Supplementary Data 1

Description: Amino acid frequencies in the consensus sequence of prestin.

The table shows the amino acid sequence of WT hPres, , which consists of the most conserved amino acids in all sequences used for consensus calculation, and their frequencies. The FWT column shows the extents of the conserved amino acids of the wild-type sequence (shown in WT 5 column) in all of the sequences used for the consensus calculation. The F_{Cons} column shows the frequency of the most conserved amino acids in the calculated sequences. The F_{Con}/F_{WT} column shows the ratio of the frequencies of WT and the most conserved sequence, and this value is used as the basis for determining the introduction of the thermo-stabilizing mutations: the higher the F_{Con}/F_{WT}, the greater preference to change the amino acids from WT to the most conserved 10 sequence.

File name: Supplementary Data 2

Description: The codon-optimized DNA sequence of thermostabilized prestin ($Pres^{TS}$).

The codon-optimized DNA sequence of $Pres^{TS}$.

File name: Supplementary Movie 1

Description: Rigid-body domain movement deduced from the Cl^{-} - vs. salicylate-bound $Pres^{TS}$ structures.

The salicylate-bound and Cl^{-} -bound structures are overlayed with the gate domains used as superimposition references. The anion binding site is indicated by a circle. See also Fig. 3c.

File name: Supplementary Movie 2

Description: Salicylate-induced elongation of mouse OHCs.

Salicylate (1.5 mM) was added to the bath solution at time zero. The total filming time is 116 seconds. OHC elongation was quantified to be 3.2±0.7% (mean±SD, n=11).

File name: Supplementary Movie 3

Description: Shortening of OHCs after salicylate elimination.

Salicylate (1.5 mM) was perfused away starting at time zero. The total filming time is 200 seconds. OHC shortening was quantified to be -2.9±1.5% (mean±SD, n=12).
