# Supplementary material for: Cryo-EM structures of thermostabilized prestin provide mechanistic insights underlying outer hair cell electromotility
Source: Nat Commun. 2022 Oct 20;13:6208. doi: 10.1038/s41467-022-34017-x (PMC9584906; doi:10.1038/s41467-022-34017-x)
Supplement: Supplementary file 9 — Reporting Summary [file 41467_2022_34017_MOESM9_ESM.pdf]

nature portfolio

Corresponding author(s): Tomohiro Nishizawa, Kazuaki Homma and Osamu Nureki

Last updated by author(s): Oct 1, 2022

Reporting Summary

Nature Portfolio wishes to improve the reproducibility of the work that we publish. This form provides structure for consistency and transparency in reporting. For further information on Nature Portfolio policies, see our Editorial Policies and the Editorial Policy Checklist.

Statistics

For all statistical analyses, confirm that the following items are present in the figure legend, table legend, main text, or Methods section.

n/a	Confirmed	
☐	☒	The exact sample size (*n*) for each experimental group/condition, given as a discrete number and unit of measurement
☐	☒	A statement on whether measurements were taken from distinct samples or whether the same sample was measured repeatedly
☐	☒	The statistical test(s) used AND whether they are one- or two-sided<br>*Only common tests should be described solely by name; describe more complex techniques in the Methods section.*
☒	☐	A description of all covariates tested
☒	☐	A description of any assumptions or corrections, such as tests of normality and adjustment for multiple comparisons
☐	☒	A full description of the statistical parameters including central tendency (e.g. means) or other basic estimates (e.g. regression coefficient) AND variation (e.g. standard deviation) or associated estimates of uncertainty (e.g. confidence intervals)
☐	☒	For null hypothesis testing, the test statistic (e.g. *F*, *t*, *r*) with confidence intervals, effect sizes, degrees of freedom and *P* value noted<br>*Give P values as exact values whenever suitable.*
☒	☐	For Bayesian analysis, information on the choice of priors and Markov chain Monte Carlo settings
☒	☐	For hierarchical and complex designs, identification of the appropriate level for tests and full reporting of outcomes
☒	☐	Estimates of effect sizes (e.g. Cohen's *d*, Pearson's *r*), indicating how they were calculated

Our web collection on statistics for biologists contains articles on many of the points above.

Software and code

Policy information about availability of computer code

Data collection	Serial EM 3.7.4, Amber ver.16, NAMD 2.12b1 for Linux-x86_64-MPI, VMD for LINUXAMD64 version 1.9.3, jClamp 23.1.1
Data analysis	RELION-3.1, PHENIX-1.14-3260, CTFFIND4.1.13, COOT-0.9.6, Cuemol 2.2.3.443, Prism 8.0.0, Origin7, pytc, UCSF ChimeraX (version 1.3), Pytc, cpptraj (AmberTools16)

For manuscripts utilizing custom algorithms or software that are central to the research but not yet described in published literature, software must be made available to editors and reviewers. We strongly encourage code deposition in a community repository (e.g. GitHub). See the Nature Portfolio guidelines for submitting code & software for further information.

Data

Policy information about availability of data

All manuscripts must include a data availability statement. This statement should provide the following information, where applicable:

- Accession codes, unique identifiers, or web links for publicly available datasets
- A description of any restrictions on data availability
- For clinical datasets or third party data, please ensure that the statement adheres to our policy

Cryo-EM density maps obtained in this study have been deposited in the Electron Microscopy Data Bank under the accession codes EMD-31757 (Cl−-bound), 31758 (SO42−-bound) and 31759 (salicylate-bound). Atomic coordinates obtained in this study have been deposited in the Protein Data Bank under IDs 7V73 [http://doi.org/10.2210/pdb7V73/pdb] (Cl−-bound), 7V74 [http://doi.org/10.2210/pdb7V74/pdb] (SO42−-bound) and 7V75 [http://doi.org/10.2210/pdb7V75[http://

doi.org/10.2210/pdb7V73/pdb]/pdb] (salicylate-bound). The raw images have been deposited in the Electron Microscopy Public Image Archive, under accession code EMPIAR-11199. Consensus sequence creator script code used in this study is available at [https://doi.org/10.5281/zenodo.7099885]. Source data are provided with this paper. All other data and an in-house program for electrostatic calculations are available from the corresponding authors upon reasonable request. We will make the data available upon request.

Human research participants

Policy information about studies involving human research participants and Sex and Gender in Research.

Reporting on sex and gender	*Use the terms sex (biological attribute) and gender (shaped by social and cultural circumstances) carefully in order to avoid confusing both terms. Indicate if findings apply to only one sex or gender; describe whether sex and gender were considered in study design whether sex and/or gender was determined based on self-reporting or assigned and methods used. Provide in the source data disaggregated sex and gender data where this information has been collected, and consent has been obtained for sharing of individual-level data; provide overall numbers in this Reporting Summary. Please state if this information has not been collected. Report sex- and gender-based analyses where performed, justify reasons for lack of sex- and gender-based analysis.*
Population characteristics	*Describe the covariate-relevant population characteristics of the human research participants (e.g. age, genotypic information, past and current diagnosis and treatment categories). If you filled out the behavioural & social sciences study design questions and have nothing to add here, write "See above."*
Recruitment	*Describe how participants were recruited. Outline any potential self-selection bias or other biases that may be present and how these are likely to impact results.*
Ethics oversight	*Identify the organization(s) that approved the study protocol.*

Note that full information on the approval of the study protocol must also be provided in the manuscript.

Field-specific reporting

Please select the one below that is the best fit for your research. If you are not sure, read the appropriate sections before making your selection.

☒ Life sciences ☐ Behavioural & social sciences ☐ Ecological, evolutionary & environmental sciences

For a reference copy of the document with all sections, see nature.com/documents/nr-reporting-summary-flat.pdf

Life sciences study design

All studies must disclose on these points even when the disclosure is negative.

Sample size	Sample size calculation was not performed, because we do not draw statistical conclusions to claim differences in this paper.
Data exclusions	ns In NLC data analysis, noisy data collected from cells with low membrane resistance (Rm < 150MΩ) were excluded.
Replication	In NLC recording, reproducible heterologous expression of the prestin constructs in HEK293T cells was always confirmed visually using a fluorescence microscope prior to whole-cell recording.
Randomization	Randomization was not relevant because the experiments were performed under well controlled conditions (e.g., the concentrations of ions, designs of the DNA constructs, etc.). No human or animal subjects were used in the study. Randomization is not generally used in this field.
Blinding	The constructs generated in this study were handled with simple IDs (e.g., HgWM1, HgWM2). The experimenters did not know the mutation information during data collections and analyses. This is considered blinding.

Reporting for specific materials, systems and methods

We require information from authors about some types of materials, experimental systems and methods used in many studies. Here, indicate whether each material, system or method listed is relevant to your study. If you are not sure if a list item applies to your research, read the appropriate section before selecting a response.

Materials & experimental systems

n/a	Involved in the study	
☒	☐	Antibodies
☐	☒	Eukaryotic cell lines
☒	☐	Palaeontology and archaeology
☐	☒	Animals and other organisms
☒	☐	Clinical data
☒	☐	Dual use research of concern

Methods

n/a	Involved in the study	
☒	☐	ChIP-seq
☒	☐	Flow cytometry
☒	☐	MRI-based neuroimaging

Eukaryotic cell lines

Policy information about cell lines and Sex and Gender in Research

Cell line source(s)	Sf9 cells were purchased from Thermo Fischer Scientific (cat # 11496015). HEK293S GnTI- (N-acetylglucosaminyl-transferase I-negative) cells used for structural analysis were purchased from ATCC.<br>HEK293T cells used for NLC measurement were purchased from ATCC.
Authentication	The cell lines listed above were not authenticated after purchased from ATCC.
Mycoplasma contamination	The cell lines listed above were not tested for mycoplasma contamination after purchased from ATCC.
Commonly misidentified lines<br>(See ICLAC register)	Commonly misidentified cell lines were not used in this study.

Animals and other research organisms

Policy information about studies involving animals; ARRIVE guidelines recommended for reporting animal research, and Sex and Gender in Research

Laboratory animals	Wild-type FVB/NJ mice of both sexes were used. Two sentences “Mice were group housed with food and water provided ad libitum under a 12-h light/12-h dark cycle and temperatures of 18–23°C with 40–60% humidity. OHCs were isolated from wild-type FVB/NJ mice as described previously.” are added to the method section (under “animals”) of our revised manuscript.
Wild animals	Wild animals were not used in this study.
Reporting on sex	Both male and female were used. Experimental results were sex-independent.
Field-collected samples	This study did not use samples collected from the field.
Ethics oversight	For the use of FVB/NJ mice, care and use of the animals were approved by the National Institutes of Health and the Animal Care and Use Committee at Northwestern University.

Note that full information on the approval of the study protocol must also be provided in the manuscript.
